# Data-Driven Optimization and Modelling of the Gap Bridgeability Performance of Multi-Pin Friction Stir Welded EN AW 7020-T651 Joints

**DOI:** 10.3390/ma19030544

**Published:** 2026-01-29

**Authors:** Ramin Delir Nazarlou, Pouya Zarei, Samita Salim, Michael Wiegand, Martin Kahlmeyer, Stefan Böhm

**Affiliations:** Department for Cutting and Joining Manufacturing Processes, University of Kassel, 34125 Kassel, Germany

**Keywords:** friction stir welding, multi-pin, gap bridgeability, modelling, weak area percentage

## Abstract

Friction stir welding (FSW) of high-strength aluminum alloys, including EN AW 7020-T651, encounters significant challenges under weld line gap conditions, leading to compromised joint integrity. This study develops a predictive, data-driven framework to assess and optimize the gap bridgeability performance of FSW joints with weld line gaps ranging from 0 to 4 mm in 2 mm thick plates. A structured experimental matrix was implemented, systematically varying rotational speed, welding speed, axial force, and tool shoulder diameter. To promote stable material flow and consistent weld quality under varying gap conditions, a multi-pin tool was employed throughout the welding trials. This configuration supported defect-free weld formation across a broad process window and contributed to improved weld soundness under gap conditions. Weld quality was evaluated using a comprehensive, multi-criteria approach that required (i) defect-free joints verified by visual and cross-sectional (metallographic) inspection, (ii) an ultimate tensile strength of at least 230 MPa, and (iii) a novel metric termed weak area percentage (WAP). Derived from micro-hardness mapping, WAP quantified the proportion of the heat-affected zone (HAZ) exhibiting hardness below 96 HV, providing a more robust and spatially sensitive measure of mechanical integrity than conventional average hardness values. Two machine learning models, Logistic Regression and Random Forest, were trained to classify weld acceptability. The Random Forest model demonstrated superior performance, achieving 92.5% classification accuracy and an F1-score of 0.90. Feature importance analysis identified the interaction terms “welding speed × gap size” and “rotational speed × gap size” as the most influential predictors of weld quality.

## 1. Introduction

Lightweight and corrosion-resistant aluminum alloys are widely used in structural applications across transportation, shipbuilding, and aerospace sectors [1]. Among them, the heat-treatable 7xxx series offers high specific strength, excellent fatigue resistance, and reliable performance under cyclic loading, making EN AW 7020-T651 a preferred choice for demanding applications [2]. However, 7xxx alloys, like other precipitation-strengthened series such as 2xxx and 6xxx, are difficult to weld using conventional fusion-based methods due to their narrow solidification range and high susceptibility to hot cracking [3]. As a result, they are often considered non-weldable by arc or beam welding techniques, restricting their broader application in welded assemblies [4,5].

Solid-state welding methods, particularly friction stir welding (FSW), offer a promising alternative. In this process, a rotating, non-consumable tool is plunged into the interface between two adjoining work pieces, generating frictional heat and plastic deformation that consolidate the material into a sound joint without melting [6]. This technique eliminates fusion-based welding-related defects such as hot cracking, porosity, and solidification shrinkage. FSW typically produces a fine-grained, recrystallized stir zone and, when properly controlled, can yield high joint efficiencies, reduced residual stress, and enhanced corrosion resistance, making it ideal for high-performance structural aluminum applications [1,7].

Beyond thermal management, the geometry of the welding tool plays a decisive role in weld quality [8]. Conventional single-pin tools are widely used due to their simplicity and effectiveness under standard conditions. However, their ability to maintain stable and uniform material flow declines in more demanding cases, particularly at higher welding speeds or when weld line gaps were applied [9]. To overcome these limitations, recent studies have explored alternative tool configurations. Multi-pin tool designs, in particular, introduce additional stirring features that enhance plastic flow, promote induced secondary flow fields, and improve mixing within the weld zone. These secondary flow fields facilitate the lateral redistribution of softened material, which is especially critical in gap-affected root regions, thereby reducing internal defects such as incomplete root welds and the characteristic Lazy-S interface [10,11,12]. Their advantages are especially apparent in gap-affected joints, where uniform flow is critical to defect-free weld formation. The implementation of multi-pin tool geometries in FSW requires careful consideration of tool manufacturing accuracy, pin alignment, and process stability. Compared to conventional single-pin tools, multi-pin designs impose stricter requirements on tool rigidity, concentricity, and wear resistance to ensure uniform load sharing and stable material flow. In industrial applications, these requirements are primarily justified in cases where enhanced material transfer, improved gap bridgeability, or extended process windows are required, particularly for thin-gauge and high-strength aluminum alloys. When these conditions are met, multi-pin tools can be implemented within existing FSW systems without fundamental changes to machine architecture, provided that appropriate tool design and process control strategies are employed. Beyond tool design, the selection and interplay of process parameters significantly affect weld quality and gap bridgeability. Variables such as welding speed, rotational speed, axial force, and plunge depth together define the thermomechanical cycle, influencing heat input, material flow mechanisms, and macro/microstructural evolution. In precipitation-strengthened alloys, this operational window is particularly narrow, making the process highly sensitive to even minor deviations in input conditions [13]. Achieving consistent and defect-free welding under such conditions requires a careful balance of thermal and mechanical inputs. Due to the complex and coupled nature of these parameters, single-factor experimentation is insufficient. Accordingly, multivariate and data-driven approaches are required to capture the interactions governing joint formation, especially in the presence of weld line gaps.

The increasing complexity of FSW, particularly in high-strength aluminum alloys, has prompted widespread adoption of optimization and data-driven modelling techniques to enhance weld quality and process reliability. Classical approaches such as Taguchi design, factorial experiments, and Response Surface Methodology (RSM) have been commonly employed to determine optimal parameter combinations for maximizing tensile strength, improving surface finish, and ensuring proper material flow [14,15]. While these methods offer valuable empirical insights, they often fall short in capturing non-linear, multi-variable interactions, particularly within the narrow process windows typical of precipitation-hardened alloys. To overcome these limitations, machine learning (ML) methods have gained significant traction in FSW research. Models such as artificial neural networks (ANN), Random Forests (RFs), support vector machines (SVM), and gradient boosting trees (GBT) have been successfully applied to predict weld performance outcomes, including tensile strength, joint efficiency, defect formation, and micro-hardness [16,17,18,19].

More recently, data-driven and deep learning frameworks have been extended to friction stir welding of both similar and dissimilar material systems, where experimentally validated neural network-based models have demonstrated strong capability in predicting weld quality and mechanical properties across varying joint configurations [20].

For AA7xxx-series alloys, data-driven modelling has already been explored for both similar and dissimilar FSW joints. For example, ANN-based models have been reported for predicting mechanical responses in AA7020 FSW joints, demonstrating good agreement between predicted and experimental tensile-shear performance [21]. In AA7075 FSW, comparative ML frameworks have been proposed using ANN, support vector regression (SVR), and Random Forest regression (RFR) to relate process variables to mechanical and/or process responses and to support parameter optimization [22]. Recent AA7075-focused studies have also employed ensemble learning, including gradient boosting and extreme gradient boosting (XGBoost), to predict tensile and related mechanical properties, reinforcing the suitability of boosting-based models for capturing non-linear interactions in FSW datasets [23]. Additionally, predictive modelling efforts for AA7075 FSW have benchmarked learning-based approaches against methods such as SVM, indicating the growing use of ML as a practical complement to conventional regression-based optimization [24].

These models not only offer improved predictive accuracy over traditional regression techniques but also enable parameter sensitivity analysis and support multi-objective decision-making. Despite this progress, most ML applications to date assume ideal joint-line conditions and focus on fully contacting joints, treating the weld line geometry as invariant rather than as a controlled process variable. As a result, the gap dimension is rarely included explicitly in data-driven models, and the gap-induced changes in heat input, material flow behaviour, and defect formation mechanisms remain largely unexplored. Consequently, the literature lacks sufficient consideration of non-zero joint gaps, despite their practical relevance in real-world assemblies. Weld quality prediction efforts have largely focused on global mechanical properties, with tensile strength serving as the primary performance indicator. While average micro-hardness is occasionally used to supplement these models, it fails to capture localized thermal softening in the heat-affected zone (HAZ), a region typically more susceptible to failure. This study addresses the lack of spatially sensitive quality metrics by introducing the weak area percentage (WAP), which is a novel, physically grounded indicator that quantifies the proportion of the weld cross-section falling below a critical hardness threshold. By incorporating the spatial distribution of hardness, WAP reflects the combined influence of thermal history and material flow, enabling a more targeted assessment of mechanical vulnerability.

In the context of the present study, gap bridgeability is defined operationally as the ability of the FSW process to produce a defect-free and mechanically acceptable joint for a given combination of process parameters and weld line gap. Rather than being expressed as a closed-form mathematical function, gap bridgeability is evaluated through a classification-based framework that determines whether a specific gap size can be successfully bridged under defined thermomechanical conditions. The maximum acceptable gap is therefore identified implicitly as the largest gap for which welds satisfy the adopted quality criteria, including internal soundness, minimum tensile strength, and bounded localized hardness degradation. Similar experimental and operational definitions of gap bridgeability have been adopted in prior FSW studies, where gap limits are determined empirically due to the strongly coupled and non-linear nature of heat generation, material flow, and defect formation.

Gap bridgeability remains a critical challenge in the FSW of aluminum, particularly in thin-gauge structural applications. In industrial practice, perfect edge alignment is rarely achieved. Tolerances in machining, heat-treatment distortion, and geometric complexity often lead to joint-line openings ranging from 0.5 to 3 mm [10,25]. Given the occurrence of these gaps, the plasticised material must flow not only around the tool pin, but also laterally to fill the void. If material flow is inadequate, defects such as tunnelling, free voids, and incomplete root consolidation may develop discontinuities that compromise static strength and fatigue life. Although various strategies such as modified pin profiles and dual-rotation tool systems have been proposed to reduce these defects, a standardized process window for reliable gap bridging in aluminum sheets below 4 mm thickness has yet to be established.

The present study aims to address this gap by investigating friction stir welded EN AW 7020-T651 butt joints produced across a broad range of process parameters and with controlled weld line gaps of up to 4 mm. A multi-pin tool configuration is employed to promote stable material flow under gap-affected conditions. A data-driven methodology is used to evaluate weld quality and define the operational limits of gap bridgeability. In addition, a novel spatially sensitive quality metric, the WAP, is introduced to quantify localized hardness degradation within the weld cross-section. By explicitly incorporating joint gaps and spatial hardness information into the modelling framework, this work establishes a predictive basis for selecting robust FSW parameters capable of producing defect-free joints in high strength aluminum structures.

## 2. Methodology

In the present investigation, 2 mm thickness EN AW 7020 aluminum alloy sheets in the T651 temper condition, with dimensions of 210 mm × 60 mm, were used as the base material. The nominal chemical composition of the alloy, as specified by DIN EN 485-2 [26], is provided in Table 1.

**Figure 1:**
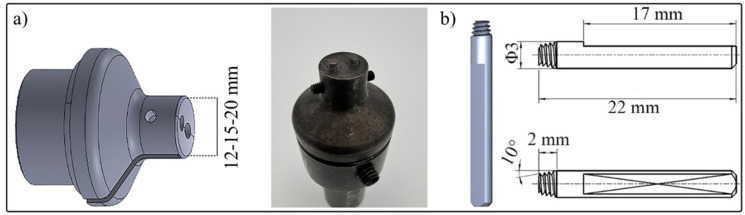
(**a**) FSW tool CAD model and tool shape. (**b**) Pin CAD model and geometry [11].

FSW was performed in a butt joint configuration along the rolling direction of the sheets using a five-axis CNC-controlled FSW machine (PTG Heavy Industries, Elland, UK) operating under force control mode. This setup enabled precise regulation of process parameters and tool path during welding. The welding tool and pins were fabricated from heat-treated H13 tool steel with a final hardness of 52 HRC and featured a tapered, threaded, multi-pin (2-pin) geometry with a 3 mm pin diameter. The pin length and pin depth were 1.48 mm and 1.64 mm, respectively. Moreover, three different shoulder diameters; 12 mm, 15 mm, and 20 mm were used to investigate the effect of shoulder size on weld quality and gap bridgeability. The tool shape and CAD model of the employed tool are shown in Figure 1.

The experimental matrix was designed to explore a wide and practical range of process conditions relevant to FSW of EN AW 7020-T651 joints (Table 2). Parameter levels were selected based on a critical review of previous studies on 7xxx-series aluminum alloys and input from industrial partners experienced in welding similar structures. The aim was to capture both low and high thermal input ranging from low-heat conditions caused by fast welding speed and low rotation speed (cold welding) to high-heat setups involving slow welding speed and high rotation speed (hot welding) in order to evaluate weld quality and identify defect formation mechanisms under challenging but realistic conditions.

Welds were produced by systematically varying five primary parameters: tool rotational and welding speed, axial force, shoulder diameter, and weld line gap. Six welding speed combinations were selected to represent progressive changes in heat input: 800 rpm–500 mm/min, 1000 rpm–500 mm/min, 1200 rpm–800 mm/min, 1400 rpm–800 mm/min, 1800 rpm–1100 mm/min, and 2000 rpm–1100 mm/min. Axial forces were varied at three levels 8, 10, and 12 kN reflecting typical vertical loading used in FSW. Weld line gaps were introduced at six levels: 0, 1.0, 2.0, 2.5, 3.0, and 4.0 mm. These gap values were selected to reflect typical joint-line openings encountered in industrial practice, arising from machining tolerances, heat-treatment distortion, and edge preparation variability. Industry reports and internal production data indicate that gaps in the range of approximately 1–3 mm are common in thin-sheet aluminum structures. The intermediate value of 2.5 mm was therefore included as a representative worst-case condition close to the upper tolerance limit, while the 4.0 mm gap was used to deliberately probe the extreme boundary of gap bridgeability. Each rotational-welding speed condition was combined with all variations in axial force, shoulder size, and gap size. This structured matrix allowed for comprehensive evaluation of the interactions between thermal-mechanical inputs and joint configuration, providing a strong basis for the microstructural analysis and data-driven modelling described in the following sections. The experimental dataset comprised all welds produced within the structured parameter matrix, with each data point corresponding to an independent welding condition defined by a unique combination of process parameters and weld line gap. All welds were evaluated and classified as “Acceptable” (OK) or “Not Acceptable” (N-OK) based on the multi-criteria quality definition described in Section 2.2. The resulting dataset exhibited a moderate class imbalance, with non-acceptable welds occurring more frequently than acceptable ones. This dataset was used in its entirety for model development, and shuffle-based 5-fold cross-validation was employed to ensure robust performance estimation and to mitigate overfitting risk.

Weld quality was evaluated using a structured two-stage protocol consisting of visual inspection followed by mechanical and macro structural analysis. Visual inspection was carried out immediately after welding to identify surface defects. For metallographic preparation, samples were mounted, ground with silicon-carbide paper up to 2500 grit, and polished to a mirror finish in accordance with DIN EN ISO 17639 [27]. Keller’s reagent was subsequently applied to reveal macro structural features. Internal soundness was examined using a DM2700 optical microscope (Leica Microsystems GmbH, Wetzlar, Germany), focusing on the detection of tunnelling, free voids, and incomplete root welding. Welds with any of these internal defects were marked as “N-OK” in the internal inspection records. Nevertheless, their corresponding tensile and hardness data were retained to serve as negative examples for subsequent data-driven modelling. Mechanical testing was conducted on welds that passed visual inspection using a Zwick/Roell Z100 universal testing machine (Zwick GmbH & Co. KG, Ulm, Germany), following the specifications of DIN EN ISO 4136 [28]. Transverse tensile specimens with a gauge section centred on the weld line were tested under displacement control at a strain rate of 0.0067 s^−1^. Load and displacement were recorded at 10 mm intervals. A weld was classified as mechanically acceptable if its ultimate tensile strength (UTS) reached or exceeded 230 MPa, corresponding to approximately 72% joint efficiency relative to the 320 MPa base-material strength. This threshold lies within the commonly reported FSW efficiency range of 67% to 94% for aluminum alloys, and was confirmed by our preliminary testing, in which lower-strength welds consistently exhibited internal discontinuities [29,30].

### 2.1. Weak Area Percentage: A New Approach to Post-Weld Assessment in FSW

In industrial settings, weld quality is typically verified through destructive tensile testing. While effective, this approach is time-consuming, costly, and statistically limited, as only a small number of specimens can be tested from each batch. As a faster and more spatially resolved alternative, hardness mapping offers valuable insight into local mechanical properties across the weld cross-section. However, most studies reduce this detailed dataset to a single scalar value, typically the average Vickers hardness within the stir zone [31,32]. This simplification fails to reflect the actual failure-sensitive area of the weld.

In precipitation-hardened aluminum alloys, failure rarely initiates in the stir zone. Instead, it commonly occurs in the thermally softened HAZ, where localized microstructural degradation can reduce hardness by 20–30% relative to the nugget zone. As a result, average hardness values often obscure critical variations in weld quality. To address this limitation, the present study introduces a more spatially sensitive and physically meaningful metric: The weak area percentage. WAP is defined as the fraction of the mapped weld area with Vickers hardness below a critical threshold of 96 HV, which corresponds approximately to a 0.2% offset yield strength of 230 MPa in EN AW 7020-T651. This threshold reflects the point at which yield strength begins to deteriorate sharply, based on prior studies of 7xxx series alloys [29,30]. While conventional one-dimensional micro-hardness profiles across the weld can be derived from the dataset, the present study adopts a spatial hardness mapping approach to better capture localized and asymmetric softening effects that are critical for weld quality classification and data-driven modelling. The selected hardness threshold of 96 HV is not intended to represent a precise local failure limit, but rather a physically grounded indicator associated with the onset of significant strength degradation in precipitation-hardened aluminum alloys. Establishing a direct, point-wise correlation between local hardness and local yield strength or ultimate tensile strength in friction stir welded EN AW 7020-T651 joints is challenging due to spatially heterogeneous microstructures, residual stress effects, and non-uniform stress states during mechanical loading. Instead, the adopted threshold is based on established empirical observations reported in the literature, where hardness values below approximately 75–80% of the base-material hardness are consistently linked to pronounced mechanical softening in the heat-affected zone. Sensitivity checks conducted during model development indicated that moderate variations in both the hardness threshold and the WAP limit did not alter weld classification trends or the relative importance of process parameters, confirming the robustness of the proposed metric.

To quantify WAP, Vickers micro-hardness measurements were carried out using an automated testing system (KB30, KB Prüftechnik GmbH, Hochdorf-Assenheim, Germany). Indentations were made at 0.5 mm intervals along three horizontal lines across the weld cross-section; one at mid-thickness and two at ±0.75 mm offsets, yielding 120 data points per weld. These values were interpolated onto a 500 × 500 radial basis grid using a custom Python 3.13.7 script developed specifically for this study. The script implemented radial basis function (RBF) interpolation to generate a continuous hardness field across the weld cross-section. The interpolation was used solely to estimate area fractions between measurement points and does not alter the underlying hardness distribution. A grid cell was flagged as part of the “weakened area” if its interpolated hardness fell below 96 HV.

WAP is defined as

(1) $$WAP=\frac{A_{HV}<96}{A_{mapped}}\times 100$$

where $A_{HV}<96$ is the total area of cells below the 96 HV threshold, and $A_{mapped}$ is the total area encompassed by the interpolated hardness map, excluding masked regions outside the convex hull of the indent array.

### 2.2. Predictive Modelling Methodology

To consolidate the multi-stage weld quality evaluation into a single outcome variable, a binary label termed “Acceptable Weld” was defined. This strict binary definition was intentionally adopted to reflect industrial acceptance criteria, where failure in any single quality aspect renders the joint unacceptable. A weld was classified as Acceptable (OK) only when all of the following criteria were simultaneously satisfied:

(i) The weld passed both visual and internal inspections (marked “OK” in the final check);
(ii) The measured UTS was at least 230 MPa;
(iii) The WAP did not exceed 12%.

If any of these conditions were not met, the weld was labelled as “Not Acceptable” (N-OK). Then, five process variables (tool rotational speed, welding speed, axial force, weld line gap, and shoulder diameter) served as input features. The rotational–welding speed pair was split into separate columns to retain numerical interpretability. To capture interaction effects, second-degree polynomial features were generated using the polynomial features class in scikit-learn, restricted to interaction-only terms to prevent unnecessary model complexity. The resulting design matrix contained 20 predictors. All pre-processing steps, including polynomial feature generation and feature scaling, were applied separately within each training fold during cross-validation to prevent information leakage. All features were subsequently standardized to zero mean and unit variance using Standard Scaler to ensure compatibility between models.

Moreover, two classification algorithms were selected to balance interpretability and predictive strength [33,34]:

- Logistic Regression (LR) with L2 regularization, with the regularization strength parameter (C) tuned across a logarithmic grid within each fold.
- Random Forest (RF) with 100 decision trees using Gini impurity and bootstrap aggregation to manage overfitting.

Due to the moderately imbalanced class distribution (~1:2), a 5-fold shuffle cross-validation approach was used instead of a single train/test split. As shown in Table 3 model performance was evaluated using five standard metrics: Accuracy, Precision, Recall, F1-score, and Area Under the ROC Curve (AUC ROC). Among these, the F1-score was prioritized for model selection due to its balanced sensitivity to both false positives and false negatives.

## 3. Results and Discussions

The quality evaluation process began with a visual inspection, which served as the initial screening stage for all produced welds. This inspection aimed to identify external surface defects such as excessive flash formation, surface grooving, or other visible irregularities. Welds exhibiting any of these defects were classified as visually unacceptable and marked as “N-OK”; these were excluded from further metallographic and mechanical evaluation. Conversely, welds presenting a smooth and uniform surface appearance were deemed visually acceptable “OK” and advanced to the next phase of analysis.

**Figure 2:**
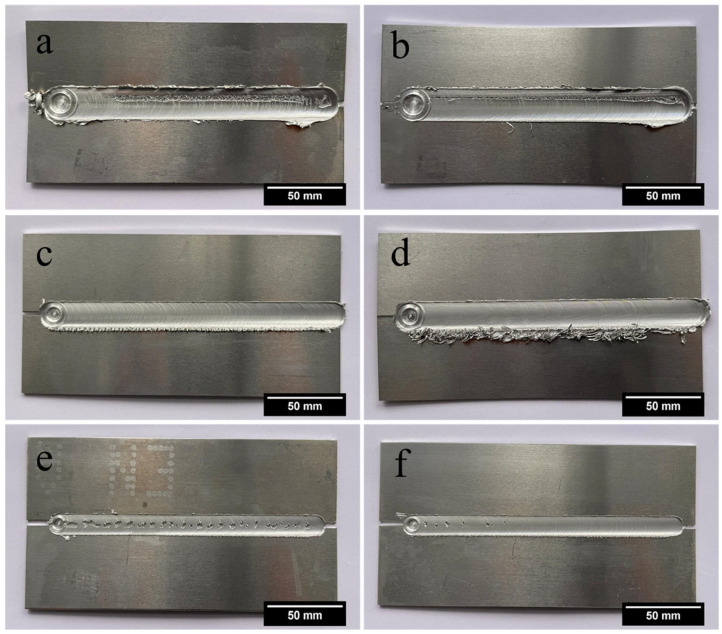
Photographs highlighting surface variations in welded samples under different welding conditions. (**a**) 1800 rpm, 1100 mm/min, gap: 3 mm, load force: 10 kN, 20 mm shoulder diameter; (**b**) 1400 rpm, 800 mm/min, gap: 2.5 mm, load force: 8 kN, 20 mm shoulder diameter; (**c**) 1200 rpm, 800 mm/min, gap: 2 mm, load force: 10 kN, 15 mm shoulder diameter; (**d**) 1000 rpm, 500 mm/min, gap: 1 mm, load force: 12 kN, 15 mm shoulder diameter; (**e**) 2000 rpm, 1100 mm/min, gap: 4 mm, load force: 8 kN, 12 mm shoulder diameter; (**f**) 1400 rpm, 800 mm/min, gap: 2.5 mm, load force: 12 kN, 12 mm shoulder diameter.

As shown in Figure 2, the surface condition of the welds was inspected in accordance with DIN EN ISO 25239-5 [35], which defines the criteria for acceptable weld surfaces in FSW. A surface was considered acceptable “OK” if it exhibited removable flash on one side and no other discernible defects.

**Figure 3:**
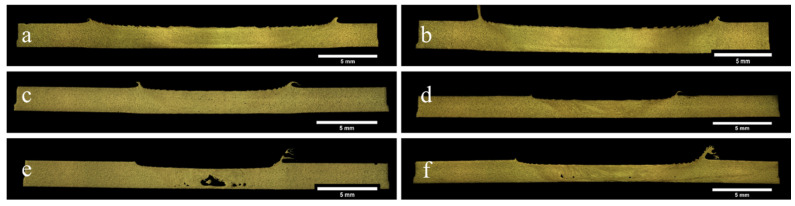
Macroscopic cross-section of exemplary welded samples. (**a**) 1800 rpm, 1100 mm/min, gap:3 mm, load force: 8 kN, 20 mm shoulder diameter; (**b**) 1200 rpm, 800 mm/min, gap:2 mm, load force: 12 kN, 20 mm shoulder diameter; (**c**) 1800 rpm, 1100 mm/min, gap: 3 mm, load force: 10 kN, 15 mm shoulder diameter; (**d**) 800 rpm, 500 mm/min, gap: 0 mm, load force: 8 kN, 15 mm shoulder diameter; (**e**) 2000 rpm, 1100 mm/min, gap: 4 mm, load force: 12 kN, 12 mm shoulder diameter; (**f**) 1800 rpm, 1100 mm/min, gap: 3 mm, load force: 10 kN, 12 mm shoulder diameter.

After visual inspection, the accepted weld samples were subjected to internal examination using metallographic cross-sectioning and optical microscopy (Figure 3). This analysis aimed to reveal subsurface imperfections such as tunnelling, voids, and incomplete root bonding defects typically linked to insufficient material flow, particularly in gap-affected joints. Several specimens that initially met visual criteria were found to contain such internal discontinuities, underscoring the limitations of surface inspection alone and highlighting the importance of a comprehensive, multi-level quality assessment.

The use of a novel multi-pin tool design significantly contributed to reducing both surface and subsurface defects across the weld parameter space. Its enhanced stirring action promoted more uniform plastic flow, which was particularly beneficial when welding joints with weld line gaps exceeding 2 mm. Under such conditions, where conventional single-pin tools typically struggle to maintain material continuity, the multi-pin configuration effectively bridged the gap and ensured more consistent weld nugget formation. This improvement was particularly evident in welds produced under high heat input regimes, where the tool geometry distributed thermal and mechanical energy more uniformly across the weld zone. As shown in Figure 3a–d, a clear trend emerged with respect to shoulder diameter. Welds produced using tools with 15 mm and 20 mm shoulder diameters exhibited superior structural integrity and internal soundness compared to those fabricated with the 12 mm shoulder. The larger shoulders facilitated greater heat generation through increased contact area, enhancing material softening and flow. This improved material consolidation and containment of the plasticized zone, particularly in joints with wider root openings. Moreover, the broader shoulder provided a stabilizing effect that helped suppress surface irregularities and root defects at higher welding speeds. The combination of multi-pin tool geometry and increased shoulder diameter emerged as a key factor in enabling defect-free weld formation, especially under demanding conditions involving larger weld line gaps and variable thermal inputs. These findings highlight the critical role of tool design parameters in establishing a stable and robust process window for friction stir welding of high-strength aluminum alloys such as EN AW 7020-T651.

**Figure 4:**
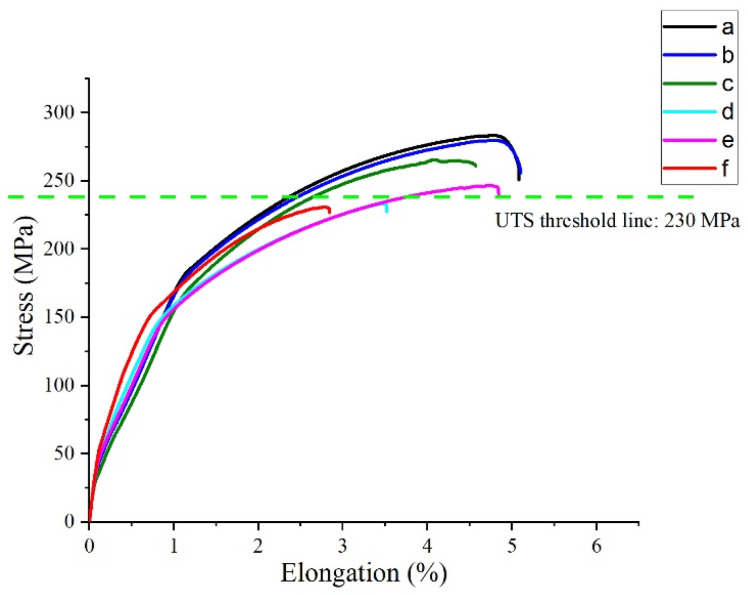
UTS results of exemplary welded samples for accepted and unaccepted welded samples under different welding conditions. (a) 800 rpm, 500 mm/min, gap: 0 mm, load force: 12 kN, 15 mm shoulder diameter; (b) 1200 rpm, 800 mm/min, gap: 2 mm, load force: 12 kN, 15 mm shoulder diameter; (c) 1200 rpm, 800 mm/min, gap: 2 mm, load force: 10 kN, 20 mm shoulder diameter; (d) 1800 rpm, 1100 mm/min, gap: 3 mm, load force: 8 kN, 12 mm shoulder diameter; (e) 1000 rpm, 500 mm/min, gap: 1 mm, load force: 12 kN, 20 mm shoulder diameter; (f) 1400 rpm, 800 mm/min, gap: 2.5 mm, load force: 10 kN, 12 mm shoulder diameter.

Mechanical performance, particularly UTS, provided further insight into the effectiveness of the applied tool and welding condition combinations. The UTS of the welded joints exhibited significant variability across the applied parameter matrix, ranging from 185 MPa to 275 MPa. This range reflects the sensitivity of mechanical performance to key process parameters such as rotational speed, welding speed, axial force, and shoulder diameter (Figure 4). As mentioned before, to ensure consistent evaluation of weld performance, the previously established UTS threshold of 230 MPa was applied as the acceptance criterion. This corresponds to approximately 72% joint efficiency relative to the 320 MPa base-material strength and serves as a realistic and technically justified benchmark for identifying mechanically sound welds in precipitation-hardened aluminum alloys.

Joints that exceeded the 230 MPa threshold typically resulted from process combinations that delivered sufficient thermal input. Notably, these included higher rotational speeds (e.g., 1200–1400 rpm), moderate welding speeds (800–1100 mm/min), and the use of larger shoulder diameters (15 mm or 20 mm). These parameters promoted enhanced plasticization and more complete material consolidation across the weld region, key conditions for achieving high tensile strength. On the other hand, joints falling below the 230 MPa threshold were generally associated with lower thermal input conditions, such as low rotational speeds (800–1000 rpm) and the use of a smaller 12 mm shoulder diameter. These settings likely led to insufficient material flow and incomplete root consolidation, conditions commonly observed under low heat input regimes in FSW of 7xxx series aluminum alloys. Such limitations can cause void formation and weak interfacial bonding, ultimately reducing mechanical reliability. Some welds achieved UTS values slightly above the 230 MPa threshold despite being produced under moderate thermal input conditions, particularly those involving 12 mm shoulder diameters and intermediate rotational speeds (1000–1200 rpm). While these joints met the minimum mechanical strength requirement, their proximity to the threshold may indicate the presence of localized soft zones or internal inconsistencies. As such characteristics are not always detectable through tensile testing alone, the WAP is introduced as a spatially resolved hardness-based metric in the following section to capture localized softening and internal inconsistencies that are otherwise obscured by global mechanical measurements.

**Figure 5:**
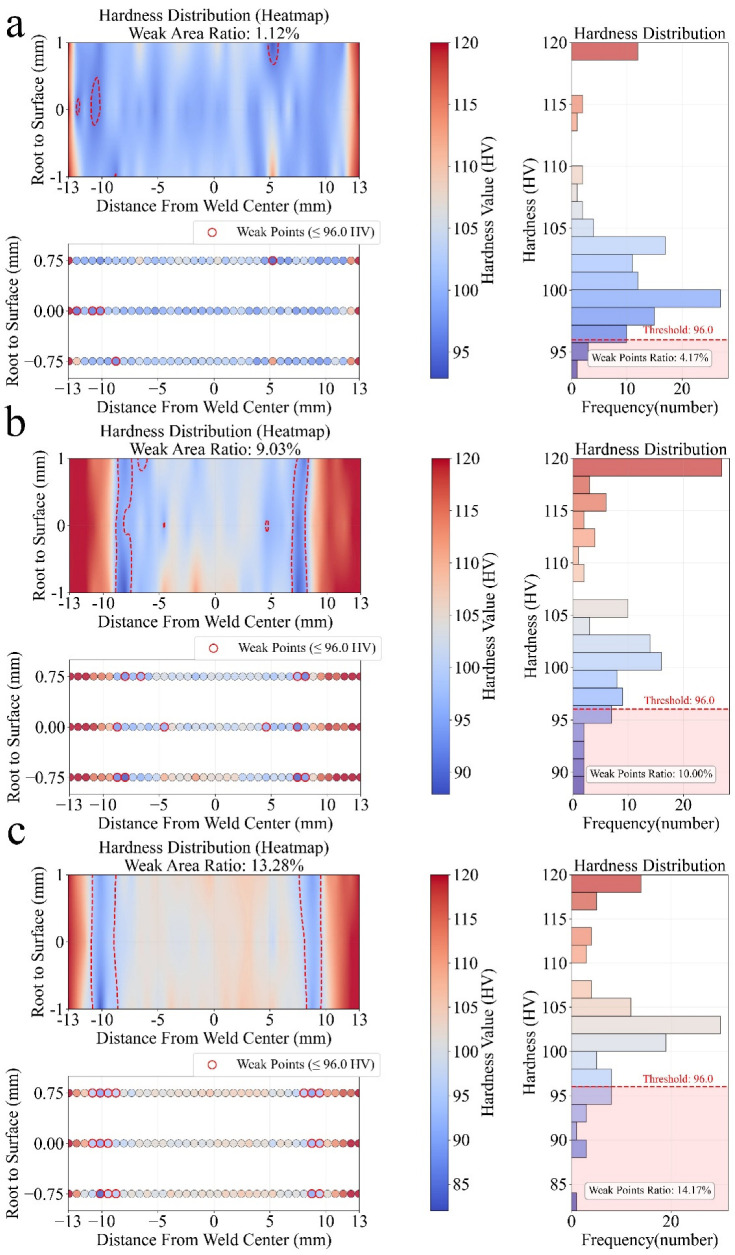
WAP results exemplary welded samples for accepted and unaccepted welded samples under different welding conditions. The dashed line highlights the WAP region. (**a**) 800 rpm, 500 mm/min, gap: 0 mm, load force: 12 kN, 15 mm shoulder diameter; (**b**) 1000 rpm, 500 mm/min, gap: 1 mm, load force: 12 kN, 20 mm shoulder diameter; (**c**) 1400 rpm, 800 mm/min, gap: 2.5 mm, load force: 10 kN, 12 mm shoulder diameter.

To complement tensile testing and provide a more spatially resolved measure of weld integrity, the WAP was employed as a post-weld evaluation metric (Figure 5). As previously described, WAP quantifies the fraction of the weld cross-section in which the local Vickers hardness falls below the critical threshold of 96 HV, corresponding to the onset of yield strength degradation in EN AW 7020-T651. This threshold, approximately 25% lower than the base-material hardness of 125 ± 3 HV, agrees with literature reporting that the tensile performance of precipitation-hardened 7xxx-series alloys deteriorates sharply once hardness drops below 75–80% of the base-material value.

To improve spatial accuracy and prevent extrapolation artefacts, a Delaunay-based convex-hull filter was applied to mask regions outside the measurement boundary and remove isolated outliers. The algorithm then calculated both point-based and area-weighted WAP values, with the latter used for all subsequent analyses. In addition, the routine automatically generated three diagnostic plots for each weld: (i) an interpolated hardness heat map with the 96 HV iso-contour, (ii) a scatterplot of indent positions highlighting sub-threshold regions, and (iii) a histogram of the hardness distribution relative to the 96 HV criterion. This automated workflow eliminated operator bias, ensured consistent processing, and preserved information on the spatial distribution of softened zones that would otherwise be lost through conventional averaging. Figure 5 shows representative hardness maps produced by the described workflow. The colour fields depict the distribution of hardness from the weld root to the surface, while red circular markers indicate indentations where hardness values fall below 96 HV. These sub-threshold regions were integrated to compute the WAP and to visualize the extent of thermally induced softening within the HAZ. Samples (a–c) correspond to welds fabricated under distinct combinations of rotational speed, traverse speed, and shoulder diameter, representing progressively different levels of thermal exposure (detailed parameters are provided in Table 2). Sample (a), produced under a low-heat condition, exhibits a narrow softened region with a WAP of ≈1%; sample (b), representing a high-heat regime, shows extensive softening from the root toward the surface (≈9% WAP); and sample (c) displays an intermediate response with localized softening mainly on the advancing side (≈13% WAP). The apparent difference in map width between samples arises from reduced indentation-grid coverage in sample (b) rather than geometric variations in weld cross-section. For clarity, only three reference depths root, mid-thickness, and surface are shown on the *y*-axis.

This comparison highlights that moderate thermal input is necessary for proper material consolidation and high UTS, whereas excessive heat input promotes over ageing and enlarges regions with hardness below 96 HV. In this sense, the thermal conditions that enable strong tensile performance do not necessarily minimize hardness loss; instead, hardness degradation increases once heat input surpasses the level required for sound consolidation, primarily due to precipitate coarsening and over ageing in the heat-affected zone. The spatially resolved hardness maps therefore provide complementary information to tensile testing and confirm WAP as a sensitive indicator of localized mechanical degradation.

**Figure 6:**
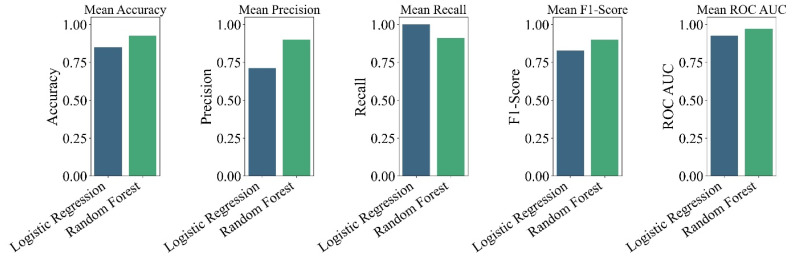
Bar-chart comparison of cross-validated metrics for Logistic Regression and Random Forest classifiers.

To support a data-driven evaluation of weld quality and enable parameter-based decision-making, two supervised machine learning models, Logistic Regression and Random Forest, were trained to predict weld acceptability based on process inputs. The binary classification target, defined in Section 2.2 consolidated visual inspection results, ultimate tensile strength (≥230 MPa), and WAP (≤12%) into a single decision label. The models were trained on five core input parameters: rotational speed, welding speed, gap size, axial force, and shoulder diameter. To capture non-linear effects and parameter interdependencies inherent to the thermomechanical FSW process, second-order polynomial interaction features were generated. All features were then standardized using Standard Scaler to ensure consistency across algorithms. Model training and evaluation were conducted using 5-fold cross-validation. Performance was assessed using Accuracy, Precision, Recall, F1-score, and Area Under the ROC Curve (AUC ROC), with the F1-score selected as the primary metric due to the class imbalance. The performance of both models is summarized in Table 4 and visualized in model performance plot (Figure 6).

**Figure 7:**
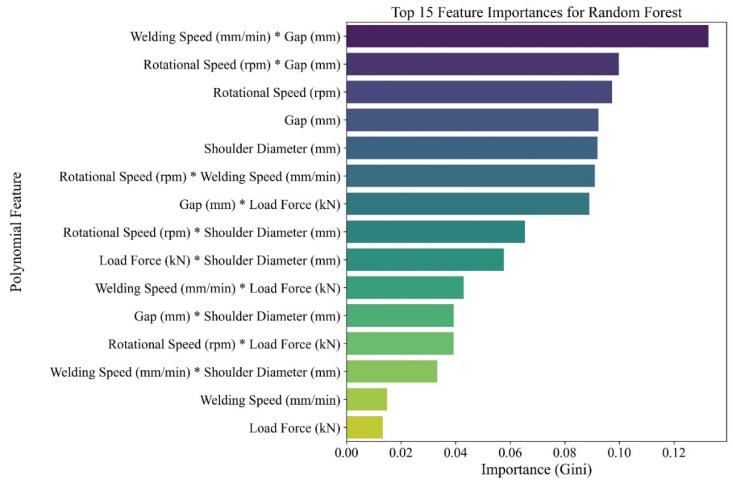
Top-15 Gini importances extracted from the trained Random Forest model; interaction terms dominate the ranking, underscoring the coupled nature of gap welding.

A clear distinction emerged between the two models. While the Logistic Regression model achieved perfect recall (1.0) meaning all acceptable welds were correctly identified it suffered from relatively low precision (0.713). This indicates a high rate of false positives, where unacceptable welds were mistakenly labelled as good, a critical risk in industrial quality assurance. This behaviour arises because the linear decision boundary of Logistic Regression is unable to fully capture the non-linear interactions between process parameters and weld quality, particularly under gap-affected conditions, leading the model to overgeneralize acceptance regions in the feature space. In contrast, the Random Forest model achieved both high precision (0.90) and recall (0.91), demonstrating excellent balance and reliability. With a superior F1-score (0.899) and the highest AUC ROC (0.972), the Random Forest classifier was selected for all subsequent analysis and predictive tasks. To gain insight into the factors driving weld quality, feature importance was extracted from the trained Random Forest model. As shown in Figure 7, while feature importances do not imply direct causality, the dominance of interaction terms rather than raw input variables is in strong agreement with established thermomechanical coupling mechanisms in friction stir welding. In particular, the most influential predictors were not the individual process parameters alone, but their interactions, as discussed below.

- “Welding Speed × Gap” and “Rotational Speed × Gap” emerged as the most influential features. These interactions reflect a clear physical basis: gap bridging success depends not just on heat input (rotational speed and feed rate), but on how that energy is distributed in relation to the void size that must be filled.
- Shoulder Diameter also ranked highly, underscoring the importance of tool geometry in governing both plasticized material volume and forging pressure during weld formation.

The model correctly identified that weld quality results from the interplay between thermal input caused by input parameters (rotational and welding speed), mechanical force, and joint configuration, rather than isolated parameter effects.

To demonstrate the practical utility of the developed model, it was applied to classify several representative parameter sets within the range covered by the experimental dataset. For illustration:

- A setup with rotational speed of 1200 rpm, welding speed of 800 mm/min, axial force of 12 kN, shoulder diameter of 15 mm, and a weld line gap of 2.0 mm was classified as “ACCEPTABLE” with a predicted class probability of 71%.
- In contrast, increasing the gap to 3.0 mm while keeping all other parameters constant resulted in a classification of “NOT ACCEPTABLE” with a predicted probability exceeding 99%, indicating a very high model confidence in rejection.

These examples illustrate the model’s capability to distinguish between low- and high-risk parameter combinations and highlight its potential use as a decision-support tool in process planning.

## 4. Conclusions

This study successfully developed and validated a machine learning framework for predicting the quality of friction stir welded EN AW 7020-T651 joints across a range of weld line gap conditions. The following key conclusions were drawn:

1. A multi-criteria definition of weld quality combining visual inspection, internal defect evaluation, mechanical strength (UTS ≥ 230 MPa), and a spatial hardness metric (WAP ≤ 12%) provides a robust and reliable assessment of joint integrity in gap-affected FSW.
2. The multi-pin tool design played a critical role in achieving defect-free welds, especially under challenging gap conditions. Its enhanced stirring action and improved material flow contributed significantly to joint consolidation, particularly in conjunction with larger shoulder diameters and higher heat input.
3. The Random Forest classifier proved to be an accurate and reliable predictive model, achieving a mean accuracy of 92.5% and an F1-score of 0.90. It outperformed Logistic Regression, which tended to over-predict acceptable welds, posing a risk in quality assurance.
4. Feature importance analysis revealed that the most influential predictors were interaction terms, particularly “Welding Speed × Gap” and “Rotational Speed × Gap”. This confirms that successful gap bridging in FSW is governed by non-linear, multi-variable relationships, and cannot be reliably optimized using single-variable adjustments.
5. The developed model has the potential to serve as a practical decision-support tool for industrial applications, enabling rapid, data-driven evaluation of process parameters and reducing reliance on trial-and-error experimentation. In its current form, the model can be used as an initial digital screening tool for proposed parameter sets within the investigated range. Further expansion of the experimental matrix is expected to improve confidence bounds and support the future derivation of a more robust gap-limit surface for design optimization.

While the present study was intentionally limited to a multi-pin tool configuration, this choice was made to ensure stable material flow and reliable gap bridging across a wide process window in thin-gauge EN AW 7020-T651 joints. The exclusive use of a multi-pin tool allowed the data-driven models to be trained under consistent geometric and thermomechanical conditions, avoiding confounding effects associated with tool geometry variation. A direct experimental comparison between single-pin and multi-pin tools, including assessment of their respective impacts on joint integrity and predictive model behaviour, represents an important and promising direction for future research. Such an extension would enable explicit quantification of tool geometry effects and further enhance the generalizability of data-driven approaches for the friction stir welding of high-strength aluminum alloys.

In addition, future work will extend the present framework by incorporating regression-based models for quantitative prediction of mechanical properties and hardness-based metrics such as UTS and WAP. This will enable continuous process window design and the derivation of gap-limit surfaces, further supporting data-driven process optimization in industrial FSW applications.

## Figures and Tables

**Table 1:** Chemical composition of 7020-T651 aluminum alloy in wt.% [11].

Element	Si	Fe	Cu	Mn	Mg	Cr	Zn	Al
wt. (%)	0.35	0.4	0.2	0.05–0.5	1–1.4	0.10–0.35	4–5	Bal.

**Table 2:** Process parameters and their levels used in the experiments.

Parameters	Levels					
	Level 1	Level 2	Level 3	Level 4	Level 5	Level 6
Tool Rotational-Welding Speed (rpm-mm/min)	800–500	1000–500	1200–800	1400–800	1800–1100	2000–1100
Weld line gap (mm)	0.0	1.0	2.0	2.5	3.0	4.0
Axial Force (kN)	8	10	12	-	-	-
Shoulder diameter (mm)	12	15	20	-	-	-

**Table 3:** Model Evaluation Metrics and their significance.

Metric	Definition	Practical Significance
Accuracy	(TP + TN)/(TP + FP + TN + FN)	Overall correctness of predictions; sensitive to class imbalance.
Precision	TP/(TP + FP)	Proportion of predicted acceptable welds that are truly acceptable; important to avoid false positives.
Recall	TP/(TP + FN)	Proportion of true acceptable welds correctly identified; helps avoid rejecting good welds.
F1-score	Harmonic mean of Precision and Recall	Balances false positives and false negatives; primary metric due to class imbalance.
AUC ROC	Area under ROC curve	Measures model’s ability to separate classes across all thresholds; closer to 1 is better.

TP = True Positives, TN = True Negatives, FP = False Positives, FN = False Negatives.

**Table 4:** Comparative performance metrics of the classification models.

Model	Accuracy	Precision	Recall	F1-Score	AUC ROC
Logistic Regression	0.849	0.713	1.000	0.827	0.926
Random Forest	0.925	0.900	0.910	0.899	0.972

## Data Availability

The original contributions presented in this study are included in the article. Further inquiries can be directed to the corresponding author.
